# Nanomaterial-Enabled Environmental Remediation and Removal of Emerging Pollutants

**DOI:** 10.3390/toxics13100810

**Published:** 2025-09-23

**Authors:** Chuanjia Jiang, Shengwei Liu, Tanapon Phenrat, Qian Sui

**Affiliations:** 1 Ministry of Education Key Laboratory of Pollution Processes and Environmental Criteria, Tianjin Key Laboratory of Environmental Remediation and Pollution Control, College of Environmental Science and Engineering, Nankai University, Tianjin 300350, China; 2 Guangdong Provincial Key Laboratory of Environmental Pollution Control and Remediation Technology, School of Environmental Science and Engineering, Sun Yat-sen University, Guangzhou 510006, China; 3 Research Unit for Integrated Natural Resources Remediation and Reclamation (IN3R), Department of Civil Engineering, Faculty of Engineering, Naresuan University, Phitsanulok 65000, Thailand; 4 State Environmental Protection Key Laboratory of Environmental Risk Assessment and Control on Chemical Process, School of Resources and Environmental Engineering, East China University of Science and Technology, Shanghai 200237, China

Rapid industrialization and urbanization in recent decades have benefited human society unprecedentedly. However, the concomitant release of various toxic and harmful substances into the environment has caused considerable ecological and human health risks [1,2,3]. In particular, emerging contaminants—including perfluorinated and polyfluoroalkyl substances (PFASs), pharmaceuticals and personal care products, organophosphate esters, and micro-/nanoplastics—have been detected across diverse environmental matrices and caused global concerns [4,5,6,7]. Therefore, enormous efforts have been devoted to the development of new technologies for removing environmental pollutants. However, there remain obstacles to high-efficiency and low-carbon environmental remediation [8], and the effective removal of emerging contaminants (e.g., PFASs and microplastics) poses significant new challenges [9,10].

The rapid development of nanotechnology has opened up new opportunities for more efficient and cost-effective pollution control and environmental remediation. A myriad of novel nanomaterials with large specific surface area and abundant surface reactive sites have been explored for the enhanced removal of various legacy and emerging pollutants via adsorption, membrane separation, catalytic oxidation/reduction/hydrolysis, and photocatalysis, etc. [11,12]. Nanomaterials can act as efficient adsorbents, not only owing to their high surface areas and well-developed pore network, but also to their nano-specific surface structures [13,14]. Synthetic membranes with confined nanostructures have demonstrated selective ion separation from complex aqueous matrices, achieving ultrahigh selectivity for a range of monovalent and divalent ions [15]. Meanwhile, the catalytic efficiency of nanomaterials for degrading pollutants can be regulated by modulating their surface atomic arrangement via facet and defect engineering [16,17]. Moreover, nanomaterials have shown tremendous potential for in situ remediation of contaminated soil and groundwater [18].

The applications and potential environmental implications of nanomaterials have emerged as one of the most active and productive research frontiers in the field of environmental science and engineering. Nevertheless, nanomaterials still face a number of challenges in practical applications, including relatively high cost, material instability, and potential environmental impact. Structural degradation, aggregation, and the loss of active sites can significantly compromise their performance [19,20]. Moreover, current applications of nanomaterials remain largely confined to laboratory or pilot-scale studies. An understanding of their realistic performance as well as environmental and health impacts remains inadequate. These uncertainties hinder the large-scale deployment of nanotechnology in environmental applications, and call for safety- and sustainability-by-design strategies [21]. Addressing these challenges requires robust interdisciplinary collaboration, spanning materials science, environmental chemistry, engineering, toxicology, ecology, and policy. Notably, emerging tools such as theoretical computation and machine learning are critical for predicting the pollutant removal performances and environmental behaviors of nanomaterials and guiding the rational design of next-generation environmental functional materials with minimized risk profiles [22,23].

This Special Issue comprises two comprehensive reviews and eight original research articles, with international authorship from six countries (Contributions 1–10). These papers highlight recent progress in the development of nanomaterials, including metal-based, carbon-based, and composite nanomaterials, for the removal of both organic pollutants (e.g., pesticides, dyes, and formaldehyde) and heavy metals. In addition to experimental investigations, we have included works to emphasize the role of computational simulation in predicting and optimizing the performance of nanomaterials. Moreover, we identify critical knowledge gaps and propose future research directions to advance the field of environmental nanotechnology. We hope that the advances and insights presented herein will inspire further innovation and foster interdisciplinary collaboration, ultimately contributing to safer, more efficient, and sustainable nanotechnology for environmental protection applications.

## Data Availability

No new data were created or analyzed in this study. Data sharing is not applicable to this article.
